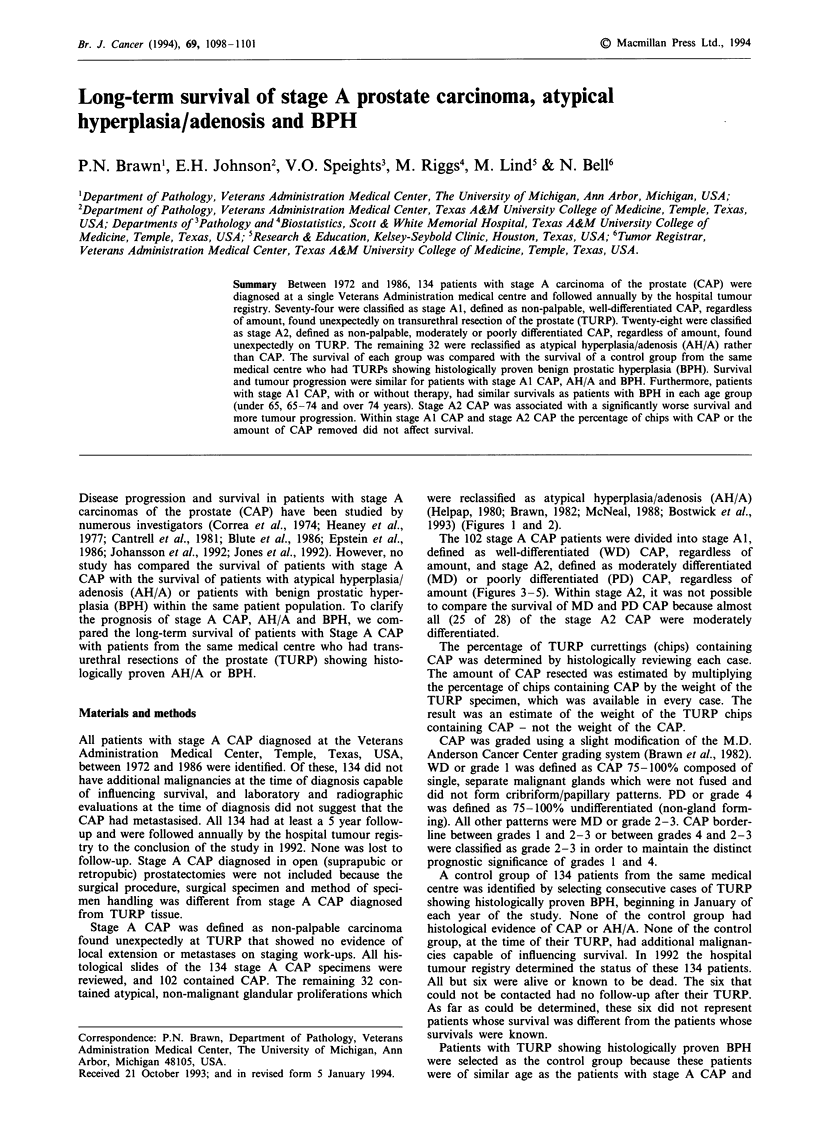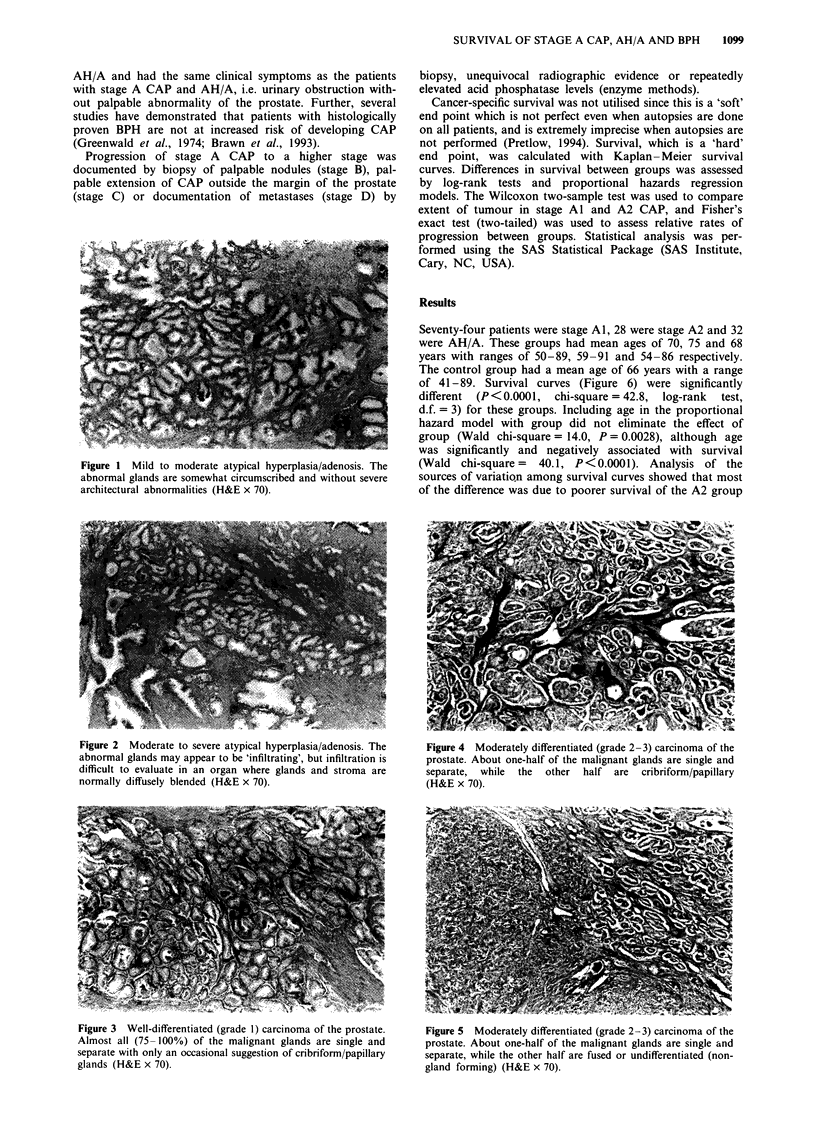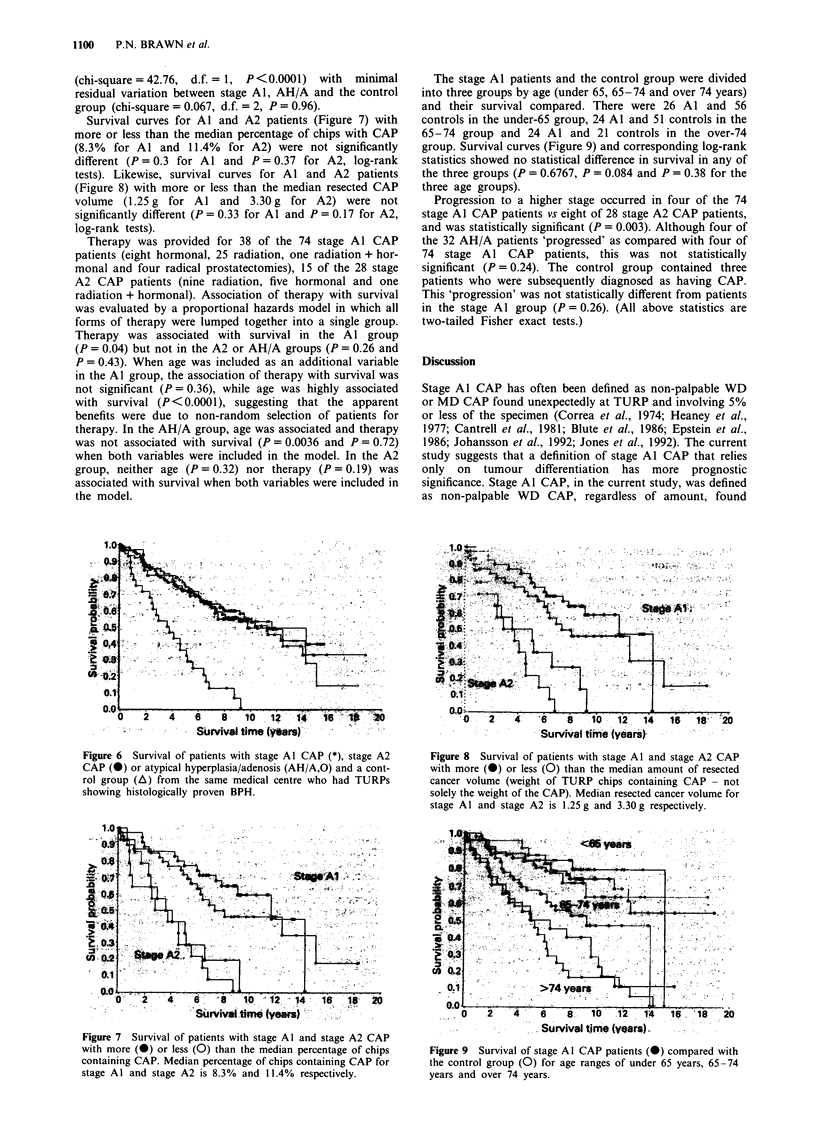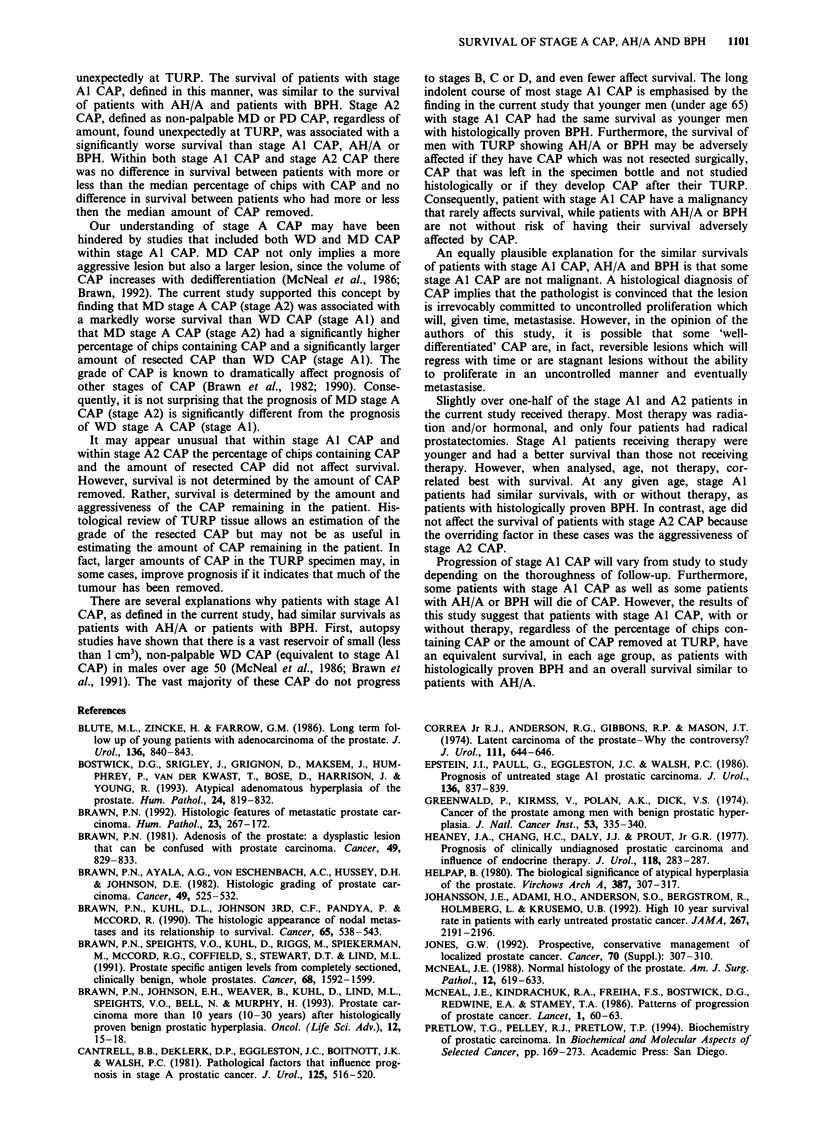# Long-term survival of stage A prostate carcinoma, atypical hyperplasia/adenosis and BPH.

**DOI:** 10.1038/bjc.1994.215

**Published:** 1994-06

**Authors:** P. N. Brawn, E. H. Johnson, V. O. Speights, M. Riggs, M. Lind, N. Bell

**Affiliations:** Department of Pathology, Veterans Administration Medical Center, Ann Arbor, Michigan 48105.

## Abstract

**Images:**


Br. J. Cancer (1994), 69, 1098-1101 © Macmillan Press Ltd., 1994

### Long-term survival of stage A prostate carcinoma, atypical hyperplasia/adenosis and BPH

P.N. Brawn[1], E.H. Johnson[2], V.O. Speights[3], M. Riggs[4], M. Lind[5] & N. Bell[6]

[1]*Department of Pathology, Veterans Administration Medical Center, The University of Michigan, Ann Arbor, Michigan, USA;* [2]*Department of Pathology, Veterans Administration Medical Center, Texas A&M University College of Medicine, Temple, Texas, USA; Departments of* [3]*Pathology and* [4]*Biostatistics, Scott & White Memorial Hospital, Texas A&M University College of Medicine, Temple, Texas, USA;* [5]*Research & Education, Kelsey-Seybold Clinic, Houston, Texas, USA;* [6]*Tumor Registrar, Veterans Administration Medical Center, Texas A&M University College of Medicine, Temple, Texas, USA.*

**Summary** Between 1972 and 1986, 134 patients with stage A carcinoma of the prostate (CAP) were diagnosed at a single Veterans Administration medical centre and followed annually by the hospital tumour registry. Seventy-four were classified as stage A1, defined as non-palpable, well-differentiated CAP, regardless of amount, found unexpectedly on transurethral resection of the prostate (TURP). Twenty-eight were classified as stage A2, defined as non-palpable, moderately or poorly differentiated CAP, regardless of amount, found unexpectedly on TURP. The remaining 32 were reclassified as atypical hyperplasia/adenosis (AH/A) rather than CAP. The survival of each group was compared with the survival of a control group from the same medical centre who had TURPs showing histologically proven benign prostatic hyperplasia (BPH). Survival and tumour progression were similar for patients with stage A1 CAP, AH/A and BPH. Furthermore, patients with stage A1 CAP, with or without therapy, had similar survivals as patients with BPH in each age group (under 65, 65-74 and over 74 years). Stage A2 CAP was associated with a significantly worse survival and more tumour progression. Within stage A1 CAP and stage A2 CAP the percentage of chips with CAP or the amount of CAP removed did not affect survival.

Disease progression and survival in patients with stage A carcinomas of the prostate (CAP) have been studied by numerous investigators (Correa *et al.*, 1974; Heaney *et al.*, 1977; Cantrell *et al.*, 1981; Blute *et al.*, 1986; Epstein *et al.*, 1986; Johansson *et al.*, 1992; Jones *et al.*, 1992). However, no study has compared the survival of patients with stage A CAP with the survival of patients with atypical hyperplasia/adenosis (AH/A) or patients with benign prostatic hyperplasia (BPH) within the same patient population. To clarify the prognosis of stage A CAP, AH/A and BPH, we compared the long-term survival of patients with Stage A CAP with patients from the same medical centre who had transurethral resections of the prostate (TURP) showing histologically proven AH/A or BPH.

### Materials and methods

All patients with stage A CAP diagnosed at the Veterans Administration Medical Center, Temple, Texas, USA, between 1972 and 1986 were identified. Of these, 134 did not have additional malignancies at the time of diagnosis capable of influencing survival, and laboratory and radiographic evaluations at the time of diagnosis did not suggest that the CAP had metastasised. All 134 had at least a 5 year follow-up and were followed annually by the hospital tumour registry to the conclusion of the study in 1992. None was lost to follow-up. Stage A CAP diagnosed in open (suprapubic or retropubic) prostatectomies were not included because the surgical procedure, surgical specimen and method of specimen handling was different from stage A CAP diagnosed from TURP tissue.

Stage A CAP was defined as non-palpable carcinoma found unexpectedly at TURP that showed no evidence of local extension or metastases on staging work-ups. All histological slides of the 134 stage A CAP specimens were reviewed, and 102 contained CAP. The remaining 32 contained atypical, non-malignant glandular proliferations which were reclassified as atypical hyperplasia/adenosis (AH/A) (Helpap, 1980; Brawn, 1982; McNeal, 1988; Bostwick *et al.*, 1993) (Figures 1 and 2).

The 102 stage A CAP patients were divided into stage A1, defined as well-differentiated (WD) CAP, regardless of amount, and stage A2, defined as moderately differentiated (MD) or poorly differentiated (PD) CAP, regardless of amount (Figures 3-5). Within stage A2, it was not possible to compare the survival of MD and PD CAP because almost all (25 of 28) of the stage A2 CAP were moderately differentiated.

The percentage of TURP currettings (chips) containing CAP was determined by histologically reviewing each case. The amount of CAP resected was estimated by multiplying the percentage of chips containing CAP by the weight of the TURP specimen, which was available in every case. The result was an estimate of the weight of the TURP chips containing CAP - not the weight of the CAP.

CAP was graded using a slight modification of the M.D. Anderson Cancer Center grading system (Brawn *et al.*, 1982). WD or grade 1 was defined as CAP 75-100% composed of single, separate malignant glands which were not fused and did not form cribriform/papillary patterns. PD or grade 4 was defined as 75-100% undifferentiated (non-gland forming). All other patterns were MD or grade 2-3. CAP borderline between grades 1 and 2-3 or between grades 4 and 2-3 were classified as grade 2-3 in order to maintain the distinct prognostic significance of grades 1 and 4.

A control group of 134 patients from the same medical centre was identified by selecting consecutive cases of TURP showing histologically proven BPH, beginning in January of each year of the study. None of the control group had histological evidence of CAP or AH/A. None of the control group, at the time of their TURP, had additional malignancies capable of influencing survival. In 1992 the hospital tumour registry determined the status of these 134 patients. All but six were alive or known to be dead. The six that could not be contacted had no follow-up after their TURP. As far as could be determined, these six did not represent patients whose survival was different from the patients whose survivals were known.

Patients with TURP showing histologically proven BPH were selected as the control group because these patients were of similar age as the patients with stage A CAP and

Correspondence: P.N. Brawn, Department of Pathology, Veterans Administration Medical Center, The University of Michigan, Ann Arbor, Michigan 48105, USA.
Received 21 October 1993; and in revised form 5 January 1994.

AH/A and had the same clinical symptoms as the patients with stage A CAP and AH/A, i.e. urinary obstruction without palpable abnormality of the prostate. Further, several studies have demonstrated that patients with histologically proven BPH are not at increased risk of developing CAP (Greenwald *et al.*, 1974; Brawn *et al.*, 1993).

Progression of stage A CAP to a higher stage was documented by biopsy of palpable nodules (stage B), palpable extension of CAP outside the margin of the prostate (stage C) or documentation of metastases (stage D) by biopsy, unequivocal radiographic evidence or repeatedly elevated acid phosphatase levels (enzyme methods).

Cancer-specific survival was not utilised since this is a 'soft' end point which is not perfect even when autopsies are done on all patients, and is extremely imprecise when autopsies are not performed (Pretlow, 1994). Survival, which is a 'hard' end point, was calculated with Kaplan–Meier survival curves. Differences in survival between groups was assessed by log-rank tests and proportional hazards regression models. The Wilcoxon two-sample test was used to compare extent of tumour in stage A1 and A2 CAP, and Fisher's exact test (two-tailed) was used to assess relative rates of progression between groups. Statistical analysis was performed using the SAS Statistical Package (SAS Institute, Cary, NC, USA).

**Figure 1** Mild to moderate atypical hyperplasia/adenosis. The abnormal glands are somewhat circumscribed and without severe architectural abnormalities (H&E × 70).

### Results

Seventy-four patients were stage A1, 28 were stage A2 and 32 were AH/A. These groups had mean ages of 70, 75 and 68 years with ranges of 50–89, 59–91 and 54–86 respectively. The control group had a mean age of 66 years with a range of 41–89. Survival curves (Figure 6) were significantly different ($P < 0.0001$, chi-square = 42.8, log-rank test, d.f. = 3) for these groups. Including age in the proportional hazard model with group did not eliminate the effect of group (Wald chi-square = 14.0, $P = 0.0028$), although age was significantly and negatively associated with survival (Wald chi-square = 40.1, $P < 0.0001$). Analysis of the sources of variation among survival curves showed that most of the difference was due to poorer survival of the A2 group

**Figure 2** Moderate to severe atypical hyperplasia/adenosis. The abnormal glands may appear to be 'infiltrating', but infiltration is difficult to evaluate in an organ where glands and stroma are normally diffusely blended (H&E × 70).

**Figure 3** Well-differentiated (grade 1) carcinoma of the prostate. Almost all (75–100%) of the malignant glands are single and separate with only an occasional suggestion of cribriform/papillary glands (H&E × 70).

**Figure 4** Moderately differentiated (grade 2–3) carcinoma of the prostate. About one-half of the malignant glands are single and separate, while the other half are cribriform/papillary (H&E × 70).

**Figure 5** Moderately differentiated (grade 2–3) carcinoma of the prostate. About one-half of the malignant glands are single and separate, while the other half are fused or undifferentiated (non-gland forming) (H&E × 70).

(chi-square = 42.76, d.f. = 1, $P<0.0001$) with minimal residual variation between stage A1, AH/A and the control group (chi-square = 0.067, d.f. = 2, $P = 0.96$).

Survival curves for A1 and A2 patients (Figure 7) with more or less than the median percentage of chips with CAP (8.3% for A1 and 11.4% for A2) were not significantly different ($P = 0.3$ for A1 and $P = 0.37$ for A2, log-rank tests). Likewise, survival curves for A1 and A2 patients (Figure 8) with more or less than the median resected CAP volume (1.25 g for A1 and 3.30 g for A2) were not significantly different ($P = 0.33$ for A1 and $P = 0.17$ for A2, log-rank tests).

Therapy was provided for 38 of the 74 stage A1 CAP patients (eight hormonal, 25 radiation, one radiation + hormonal and four radical prostatectomies), 15 of the 28 stage A2 CAP patients (nine radiation, five hormonal and one radiation + hormonal). Association of therapy with survival was evaluated by a proportional hazards model in which all forms of therapy were lumped together into a single group. Therapy was associated with survival in the A1 group ($P = 0.04$) but not in the A2 or AH/A groups ($P = 0.26$ and $P = 0.43$). When age was included as an additional variable in the A1 group, the association of therapy with survival was not significant ($P = 0.36$), while age was highly associated with survival ($P<0.0001$), suggesting that the apparent benefits were due to non-random selection of patients for therapy. In the AH/A group, age was associated and therapy was not associated with survival ($P = 0.0036$ and $P = 0.72$) when both variables were included in the model. In the A2 group, neither age ($P = 0.32$) nor therapy ($P = 0.19$) was associated with survival when both variables were included in the model.

The stage A1 patients and the control group were divided into three groups by age (under 65, 65–74 and over 74 years) and their survival compared. There were 26 A1 and 56 controls in the under-65 group, 24 A1 and 51 controls in the 65–74 group and 24 A1 and 21 controls in the over-74 group. Survival curves (Figure 9) and corresponding log-rank statistics showed no statistical difference in survival in any of the three groups ($P = 0.6767$, $P = 0.084$ and $P = 0.38$ for the three age groups).

Progression to a higher stage occurred in four of the 74 stage A1 CAP patients *vs* eight of 28 stage A2 CAP patients, and was statistically significant ($P = 0.003$). Although four of the 32 AH/A patients 'progressed' as compared with four of 74 stage A1 CAP patients, this was not statistically significant ($P = 0.24$). The control group contained three patients who were subsequently diagnosed as having CAP. This 'progression' was not statistically different from patients in the stage A1 group ($P = 0.26$). (All above statistics are two-tailed Fisher exact tests.)

### Discussion

Stage A1 CAP has often been defined as non-palpable WD or MD CAP found unexpectedly at TURP and involving 5% or less of the specimen (Correa *et al.*, 1974; Heaney *et al.*, 1977; Cantrell *et al.*, 1981; Blute *et al.*, 1986; Epstein *et al.*, 1986; Johansson *et al.*, 1992; Jones *et al.*, 1992). The current study suggests that a definition of stage A1 CAP that relies only on tumour differentiation has more prognostic significance. Stage A1 CAP, in the current study, was defined as non-palpable WD CAP, regardless of amount, found

**Figure 6** Survival of patients with stage A1 CAP (*), stage A2 CAP (●) or atypical hyperplasia/adenosis (AH/A,O) and a control group (Δ) from the same medical centre who had TURPs showing histologically proven BPH.

**Figure 7** Survival of patients with stage A1 and stage A2 CAP with more (●) or less (O) than the median percentage of chips containing CAP. Median percentage of chips containing CAP for stage A1 and stage A2 is 8.3% and 11.4% respectively.

**Figure 8** Survival of patients with stage A1 and stage A2 CAP with more (●) or less (O) than the median amount of resected cancer volume (weight of TURP chips containing CAP – not solely the weight of the CAP). Median resected cancer volume for stage A1 and stage A2 is 1.25 g and 3.30 g respectively.

**Figure 9** Survival of stage A1 CAP patients (●) compared with the control group (O) for age ranges of under 65 years, 65–74 years and over 74 years.

unexpectedly at TURP. The survival of patients with stage A1 CAP, defined in this manner, was similar to the survival of patients with AH/A and patients with BPH. Stage A2 CAP, defined as non-palpable MD or PD CAP, regardless of amount, found unexpectedly at TURP, was associated with a significantly worse survival than stage A1 CAP, AH/A or BPH. Within both stage A1 CAP and stage A2 CAP there was no difference in survival between patients with more or less than the median percentage of chips with CAP and no difference in survival between patients who had more or less then the median amount of CAP removed.

Our understanding of stage A CAP may have been hindered by studies that included both WD and MD CAP within stage A1 CAP. MD CAP not only implies a more aggressive lesion but also a larger lesion, since the volume of CAP increases with dedifferentiation (McNeal *et al.*, 1986; Brawn, 1992). The current study supported this concept by finding that MD stage A CAP (stage A2) was associated with a markedly worse survival than WD CAP (stage A1) and that MD stage A CAP (stage A2) had a significantly higher percentage of chips containing CAP and a significantly larger amount of resected CAP than WD CAP (stage A1). The grade of CAP is known to dramatically affect prognosis of other stages of CAP (Brawn *et al.*, 1982; 1990). Consequently, it is not surprising that the prognosis of MD stage A CAP (stage A2) is significantly different from the prognosis of WD stage A CAP (stage A1).

It may appear unusual that within stage A1 CAP and within stage A2 CAP the percentage of chips containing CAP and the amount of resected CAP did not affect survival. However, survival is not determined by the amount of CAP removed. Rather, survival is determined by the amount and aggressiveness of the CAP remaining in the patient. Histological review of TURP tissue allows an estimation of the grade of the resected CAP but may not be as useful in estimating the amount of CAP remaining in the patient. In fact, larger amounts of CAP in the TURP specimen may, in some cases, improve prognosis if it indicates that much of the tumour has been removed.

There are several explanations why patients with stage A1 CAP, as defined in the current study, had similar survivals as patients with AH/A or patients with BPH. First, autopsy studies have shown that there is a vast reservoir of small (less than 1 $cm^3$), non-palpable WD CAP (equivalent to stage A1 CAP) in males over age 50 (McNeal *et al.*, 1986; Brawn *et al.*, 1991). The vast majority of these CAP do not progress to stages B, C or D, and even fewer affect survival. The long indolent course of most stage A1 CAP is emphasised by the finding in the current study that younger men (under age 65) with stage A1 CAP had the same survival as younger men with histologically proven BPH. Furthermore, the survival of men with TURP showing AH/A or BPH may be adversely affected if they have CAP which was not resected surgically, CAP that was left in the specimen bottle and not studied histologically or if they develop CAP after their TURP. Consequently, patient with stage A1 CAP have a malignancy that rarely affects survival, while patients with AH/A or BPH are not without risk of having their survival adversely affected by CAP.

An equally plausible explanation for the similar survivals of patients with stage A1 CAP, AH/A and BPH is that some stage A1 CAP are not malignant. A histological diagnosis of CAP implies that the pathologist is convinced that the lesion is irrevocably committed to uncontrolled proliferation which will, given time, metastasise. However, in the opinion of the authors of this study, it is possible that some 'well-differentiated' CAP are, in fact, reversible lesions which will regress with time or are stagnant lesions without the ability to proliferate in an uncontrolled manner and eventually metastasise.

Slightly over one-half of the stage A1 and A2 patients in the current study received therapy. Most therapy was radiation and/or hormonal, and only four patients had radical prostatectomies. Stage A1 patients receiving therapy were younger and had a better survival than those not receiving therapy. However, when analysed, age, not therapy, correlated best with survival. At any given age, stage A1 patients had similar survivals, with or without therapy, as patients with histologically proven BPH. In contrast, age did not affect the survival of patients with stage A2 CAP because the overriding factor in these cases was the aggressiveness of stage A2 CAP.

Progression of stage A1 CAP will vary from study to study depending on the thoroughness of follow-up. Furthermore, some patients with stage A1 CAP as well as some patients with AH/A or BPH will die of CAP. However, the results of this study suggest that patients with stage A1 CAP, with or without therapy, regardless of the percentage of chips containing CAP or the amount of CAP removed at TURP, have an equivalent survival, in each age group, as patients with histologically proven BPH and an overall survival similar to patients with AH/A.

### References

BLUTE, M.L., ZINCKE, H. & FARROW, G.M. (1986). Long term follow up of young patients with adenocarcinoma of the prostate. *J. Urol.*, **136**, 840–843.

BOSTWICK, D.G., SRIGLEY, J., GRIGNON, D., MAKSEM, J., HUMPHREY, P., VAN DER KWAST, T., BOSE, D., HARRISON, J. & YOUNG, R. (1993). Atypical adenomatous hyperplasia of the prostate. *Hum. Pathol.*, **24**, 819–832.

BRAWN, P.N. (1992). Histologic features of metastatic prostate carcinoma. *Hum. Pathol.*, **23**, 267–172.

BRAWN, P.N. (1981). Adenosis of the prostate: a dysplastic lesion that can be confused with prostate carcinoma. *Cancer*, **49**, 829–833.

BRAWN, P.N., AYALA, A.G., VON ESCHENBACH, A.C., HUSSEY, D.H. & JOHNSON, D.E. (1982). Histologic grading of prostate carcinoma. *Cancer*, **49**, 525–532.

BRAWN, P.N., KUHL, D.L., JOHNSON 3RD, C.F., PANDYA, P. & MCCORD, R. (1990). The histologic appearance of nodal metastases and its relationship to survival. *Cancer*, **65**, 538–543.

BRAWN, P.N., SPEIGHTS, V.O., KUHL, D., RIGGS, M., SPIEKERMAN, M., MCCORD, R.G., COFFIELD, S., STEWART, D.T. & LIND, M.L. (1991). Prostate specific antigen levels from completely sectioned, clinically benign, whole prostates. *Cancer*, **68**, 1592–1599.

BRAWN, P.N., JOHNSON, E.H., WEAVER, B., KUHL, D., LIND, M.L., SPEIGHTS, V.O., BELL, N. & MURPHY, H. (1993). Prostate carcinoma more than 10 years (10–30 years) after histologically proven benign prostatic hyperplasia. *Oncol. (Life Sci. Adv.)*, **12**, 15–18.

CANTRELL, B.B., DEKLERK, D.P., EGGLESTON, J.C., BOITNOTT, J.K. & WALSH, P.C. (1981). Pathological factors that influence prognosis in stage A prostatic cancer. *J. Urol.*, **125**, 516–520.

CORREA Jr R.J., ANDERSON, R.G., GIBBONS, R.P. & MASON, J.T. (1974). Latent carcinoma of the prostate–Why the controversy? *J. Urol.*, **111**, 644–646.

EPSTEIN, J.I., PAULL, G., EGGLESTON, J.C. & WALSH, P.C. (1986). Prognosis of untreated stage A1 prostatic carcinoma. *J. Urol.*, **136**, 837–839.

GREENWALD, P., KIRMSS, V., POLAN, A.K., DICK, V.S. (1974). Cancer of the prostate among men with benign prostatic hyperplasia. *J. Natl. Cancer Inst.*, **53**, 335–340.

HEANEY, J.A., CHANG, H.C., DALY, J.J. & PROUT, Jr G.R. (1977). Prognosis of clinically undiagnosed prostatic carcinoma and influence of endocrine therapy. *J. Urol.*, **118**, 283–287.

HELPAP, B. (1980). The biological significance of atypical hyperplasia of the prostate. *Virchows Arch A*, **387**, 307–317.

JOHANSSON, J.E., ADAMI, H.O., ANDERSON, S.O., BERGSTROM, R., HOLMBERG, L. & KRUSEMO, U.B. (1992). High 10 year survival rate in patients with early untreated prostatic cancer. *JAMA*, **267**, 2191–2196.

JONES, G.W. (1992). Prospective, conservative management of localized prostate cancer. *Cancer*, **70** (Suppl.): 307–310.

MCNEAL, J.E. (1988). Normal histology of the prostate. *Am. J. Surg. Pathol.*, **12**, 619–633.

MCNEAL, J.E., KINDRACHUK, R.A., FREIHA, F.S., BOSTWICK, D.G., REDWINE, E.A. & STAMEY, T.A. (1986). Patterns of progression of prostate cancer. *Lancet*, **1**, 60–63.

PRETLOW, T.G., PELLEY, R.J., PRETLOW, T.P. (1994). Biochemistry of prostatic carcinoma. In *Biochemical and Molecular Aspects of Selected Cancer*, pp. 169–273. Academic Press: San Diego.